# Evidence for frequency‐dependent selection maintaining polymorphism in the Batesian mimic *Papilio polytes* in multiple islands in the Ryukyus, Japan

**DOI:** 10.1002/ece3.5182

**Published:** 2019-04-24

**Authors:** Kaori Tsurui‐Sato, Yukuto Sato, Emi Kato, Mitsuho Katoh, Ryosuke Kimura, Haruki Tatsuta, Kazuki Tsuji

**Affiliations:** ^1^ Center for Strategic Research Project University of the Ryukyus Okinawa Japan; ^2^ Department of Agro‐Environmental Sciences, Faculty of Agriculture University of the Ryukyus Okinawa Japan; ^3^ The United Graduate School of Agricultural Sciences Kagoshima University Kagoshima Japan; ^4^ Department of Human Biology and Anatomy, Graduate School of Medicine University of the Ryukyus Okinawa Japan

**Keywords:** female‐limited mimetic polymorphism, mimic ratio, model abundance, phylogenetic analyses, population genetics

## Abstract

Batesian mimicry is a well‐studied adaptation for predation avoidance, in which a mimetic species resembles an unpalatable model species. Batesian mimicry can be under positive selection because of the protection gained against predators, due to resemblance to unpalatable model species. However, in some mimetic species, nonmimetic individuals are present in populations, despite the benefits of mimicry. The mechanism for evolution of such mimetic polymorphism remains an open question. Here, we address the hypothesis that the abundance of mimics is limited by that of the models, leading to mimetic polymorphism. In addition, other forces such as the effects of common ancestry and/or isolation by distance may explain this phenomenon. To investigate this question, we focused on the butterfly, *Papilio polytes,* that exhibits mimetic polymorphism on multiple islands of the Ryukyus, Japan, and performed field surveys and genetic analysis. We found that the mimic ratio of *P. polytes* was strongly correlated with the model abundance observed on each of the five islands, suggesting negative frequency‐dependent selection is driving the evolution of polymorphism in *P. polytes* populations. Molecular phylogenetic analysis indicated that the southern island populations are the major source of genetic diversity, and the middle and northern island populations arose by relatively recent migration. This view was also supported by mismatch distribution and Tajima's *D* analyses, suggesting a recent population expansion on the middle and northern islands, and stable population persistence on the southern islands. The frequency of the mimetic forms within *P. polytes* populations is thus explained by variations in the model abundance rather than by population structure. Thus, we propose that predation pressure, rather than neutral forces, have shaped the Batesian mimicry polymorphism in *P. polytes* observed in the Ryukyus.

## 1 INTRODUCTION

**Figure 1:**
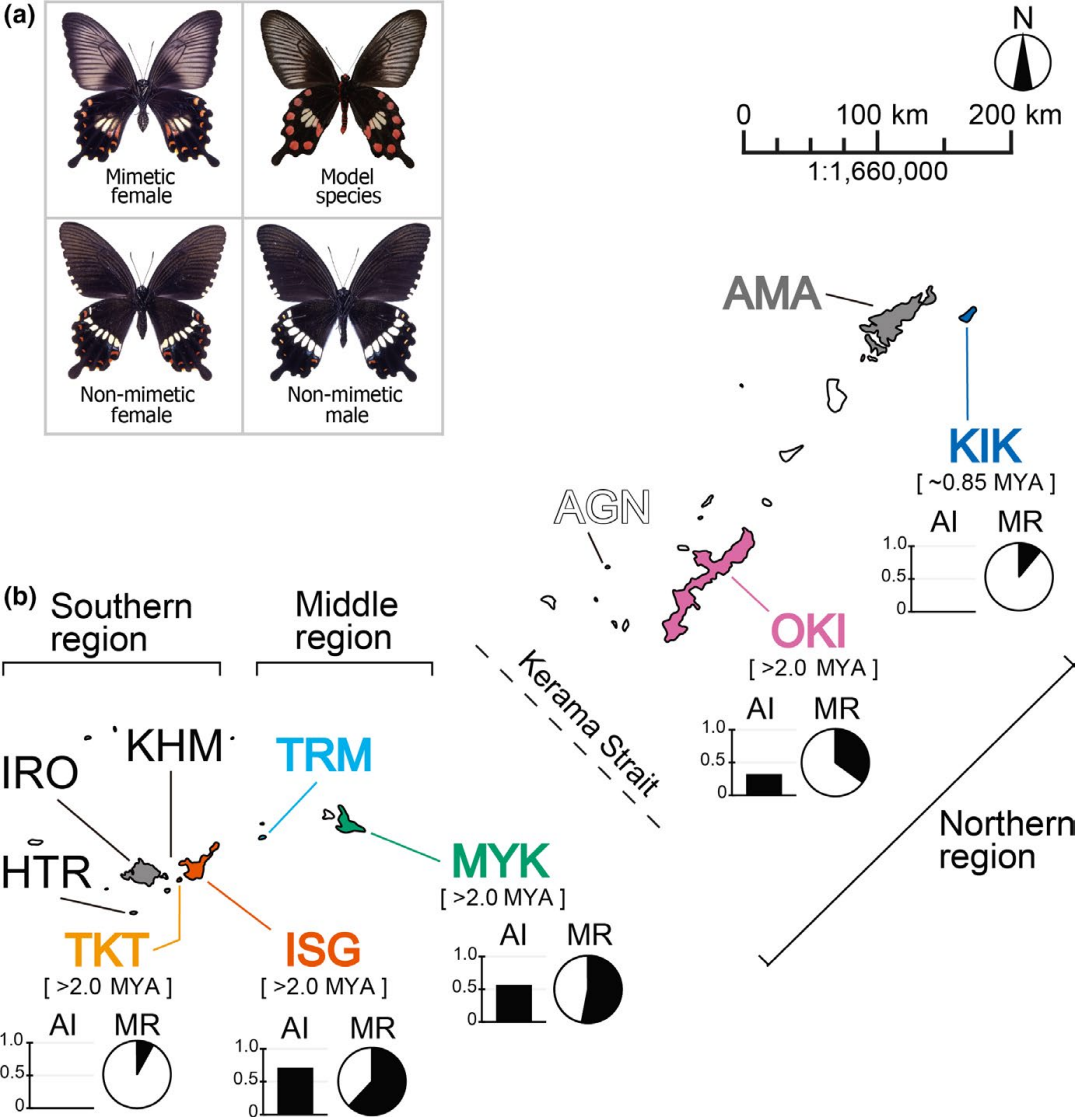
Mimicry system and sample sites in the Ryukyu Islands. (a) Mimetic females of *Papilio polytes* resemble the model species *Pachliopta aristolochiae*, whereas nonmimetic females are similar to males of *P. polytes*. (b) Map of each island, showing different characteristics in mimicry parameters AI (advantage index of Batesian mimicry) and MR (mimic ratio). Names of islands are abbreviated as follows: AGN, Aguni; AMA, Amamiooshima; HTR, Hateruma; IRO, Iriomote; ISG, Ishigaki; KHM, Kohama; KIK, Kikai; MYK, Miyako; OKI, Okinawa; TKT, Taketomi; TRM, Tarama. Island age, according to Osozawa et al. (2012), is shown in brackets

Batesian mimicry is a phenomenon in which harmless organisms resemble harmful or unpalatable species (Bates, 1862; Cott, 1940; Edmunds, 1974; Kunte, 2009; Rettenmeyer, 1970; Ruxton, Sherratt, & Speed, 2004) and is one of the most striking examples and oft‐mentioned models of Darwinian evolution. In this mimicry system, palatable prey species (mimics) receive protection from potential predators by their visual resemblance to toxic or otherwise defended species (models) (Bates, 1862; Cott, 1940; Edmunds, 1974; Kunte, 2009; Rettenmeyer, 1970; Ruxton et al., 2004). Curiously, in some butterflies, Batesian mimicry is observed in only a portion of the females (Kunte, 2009; Mallet & Joron, 1999; Wallace, 1865; Wickler, 1968). Thus, in some mimetic species, not all individuals display Batesian mimicry (see Figure 1a), despite its benefits for predator avoidance. The mechanism underlying female‐limited polymorphic mimicry has long been a matter of debate (Kunte, 2009; Mallet & Joron, 1999) and the autosomal *doublesex* (*dsx*) gene is thought to control most of the pattern variation (Clarke & Sheppard, 1972; Kunte et al., 2014; Nishikawa et al., 2015). However, it remains unclear how ecological factors and selective mechanisms shape polymorphic mimicry.

Negative frequency‐dependent selection (NFDS) has been offered as a possible mechanism underlying female‐limited polymorphic mimicry in butterflies (Barrett, 1976; Kunte, 2009; Turner, 1978), as have other hypotheses such as sexual selection and ecological–physiological trade‐offs (Burns, 1966; Cook, Vernon, Bateson, & Guilford, 1994; Ohsaki, 2005; Vane‐Wright, 1984). According to the NFDS hypothesis, the abundance of mimetic individuals is limited by the abundance of the model, because the defensive benefit of mimicry increases when the model is relatively more abundant. In other words, the advantage of mimicry decreases as the abundance of the mimics increases relative to that of the models (Barrett, 1976). Eventually, the mimic ratio (MR; the proportion of the mimic phenotype in the population) approaches an equilibrium state (Kunte, 2009), at which the fitness of mimic and nonmimic types are equal. The NFDS hypothesis predicts that this MR equilibrium corresponds to the local abundance of the model species determined by ecological factors.

On the other hand, the polymorphism can be influenced by selectively neutral processes. In other words, the mimic and nonmimic types have equal fitness independent of their frequency, and therefore, their gene frequencies in local populations change through genetic drift and migration (Ackermann & Cheverud, 2004; Wright, 1943). According to this scenario, it is expected that local populations (i.e., island populations) that are genetically closely related should show similar MRs independent of local model abundance. Therefore, it is essential to quantify the relative effects of neutral and selective processes on the persistence of polymorphism.

*Papilio polytes* L., a swallowtail butterfly that is common throughout Southeast Asia, exhibits female‐limited Batesian mimicry (Clarke & Sheppard, 1972; Katoh, Tatsuta, & Tsuji, 2017; Kunte et al., 2014; Figure 1a). Some of the females mimic other unpalatable butterflies in the family *Papilionidae*, as a defense against avian predators (Ford, 1975). The nonmimetic females resemble the males (Figure 1a). On several of the Ryukyu Islands in Japan, female *P. polytes* appear to mimic *Pachliopta aristolochiae* (Katoh et al., 2017; Uesugi, 2000). Interestingly, the MR of these *P. polytes* populations is higher on islands with greater abundance of the model species (Uesugi, 2000), implying that NFDS underlies the mimicry patterns. In addition, after the model *P. aristolochiae* became established on Miyako Island in the Ryukyus, the MR on the island increased rapidly from 1975 to 1989 (Uesugi, 2000), implying rapid local adaptation. The above observations seemed to indicate that natural selection was driving the process; however, to date no population genetic survey has been performed in *P. polytes* in the Ryukyus to test this view.

In this study, we assess the possible association between the MR of *P. polytes* and the abundance of the model species in the Ryukyu Islands. Subsequent to a previous study of these two factors performed in 1982 (Uesugi, 2000), the distribution of the model species *P. aristolochiae* has expanded (Katoh et al., 2017). Therefore, the present investigation of *P. polytes* and *P. aristolochiae* in the Ryukyu Islands provides a powerful opportunity to address the microevolution of polymorphic Batesian mimicry in *P. polytes* populations. In addition, we perform phylogenetic and population genetic analyses with mitochondrial DNA markers to examine whether neutral, nonadaptive forces such as phylogenetic constraint, isolation by distance, or demographic history can explain the MR distribution of *P. polytes* across the Ryukyu Islands. Analysis of mitochondrial markers only provides us limited genetic information of a maternally inherited locus. To improve reliability of genetic inference, analyses using multiple loci including nuclear markers are ideal. On the other hand, mitochondrial DNA analysis is reasonable for the first attempt of population genetic analyses of nonmodel organisms, because it is practically convenient. Therefore, we analyzed mitochondrial markers to obtain a first insight into the genetic structure of *P. polytes* in Ryukyu Islands in the present study.

## 2 MATERIALS AND METHODS

### 2.1 Field survey and sampling

Sample butterflies of *P. polytes* and *P. aristolochiae* were collected on the following eight islands of the Ryukyu Archipelago (the Ryukyus), southern Japan: Kikai (abbreviated as KIK), Okinawa (OKI), Miyako (MYK), Ishigaki (ISG), Taketomi (TKT), Amami (AMM), Aguni (AGN), and Tarama (TRM) (Figure 1b and Table S1). The butterflies were caught by bag net between 2014 and 2017. After the species (*P. polytes* or *P. aristolochiae*), sex, and mimic type were recorded, live all specimens were stored at −30°C in a freezer until DNA extraction (Table S1), enabling us to be certain that they were neither collected nor recorded twice. Field surveys for analysis of the association between the MR of *P. polytes* and the abundance of the model species were conducted between 2014 and 2017 (Tables S1 and S2). We excluded data collected on two days of bad weather. Namely, data collected on 17 November 2014 on Ishigaki (no butterflies collected) and 19 March 2016 on Taketomi (only one mimetic female collected) were excluded from our analyses. For genetic analysis, a portion of the *P. polytes* collected between 2014 and 2016, supplemented by additional samples collected specifically for genetic analysis between 2014 and 2016, were used (Table S1).

### 2.2 DNA extraction

Whole middle and hind legs of the butterflies were used for DNA extraction with a DNeasy Blood and Tissue Kit (Qiagen, Hilden, Germany). Samples were gently cut with hand shears and manually homogenized using disposable pestles in 180 µl of buffer ATL. The tissue homogenates were digested overnight at 55°C with 20 µl of Qiagen Proteinase K solution (600 mAU/ml) and subjected to DNA extraction. Total DNA was eluted from the spin column in 200 µl of buffer AE and stored at −30°C after confirmation of the concentration and quality using a Nanodrop 2000C (Thermo Scientific).

### 2.3 PCR amplification and parallel sequencing of mtDNA genes

To determine the mtDNA sequences of the subjects from the Ryukyu Islands, we applied long‐range PCR and shotgun sequencing using the MiSeq platform (Illumina). This approach prevented amplification of nonpreferred sequences and contamination by short nuclear pseudogenes of mitochondrial origin, which often produce misleading results in evolutionary analyses (Song, Buhay, Whiting, & Crandall, 2008). Universal primers for insect mtDNA (Simon et al., 1994) with slight modifications based on sequence alignment with closely related species, *P. machaon* (GenBank accession number: HM243594), *P. helenus* (KM244656), *P. maackii* (KC433408), *P. syfanius* (KJ396621), and *Troides aeacus* (EU625344) were used to amplify *P. polytes* mtDNA (GenBank accession number: NC_024742) (Table S3). PrimeSTAR GXL DNA Polymerase (Takara) was used, with the following PCR parameters: 30 cycles at 98°C for 10 s and 64°C for 10 min to amplify targets longer than 3 kbp. To amplify targets shorter than 3 kbp, different PCR parameters were used: 30 cycles at 98°C for 10 s, 60°C for 15 s, and 68°C for 2 min.

The PCR products were used to prepare sequencing libraries with a Nextera XT DNA Library Prep kit, which uses a transposome‐based approach, and an Index kit v2 (Illumina). To improve the fidelity of library amplification, the Nextera PCR master mix (NPM) was replaced with the same volume of KAPA HiFi HotStart ReadyMix (Kapa Biosystems). The amplified libraries, containing sequences ranging in length from 300 to 800 bp, were dissected from 1.0% L03 agarose gels (Takara) using a MinElute gel extraction kit (Qiagen), quantified by a Qubit fluorometer with a dsDNA high‐sensitivity assay kit (Thermo Scientific), and sequenced using a MiSeq Reagent Kit v2 (Illumina) to generate 2 × 250‐bp paired‐end reads (run numbers 1 to 3) and 2 × 150‐bp paired‐end reads (run numbers 4 to 8) (see Table S4).

### 2.4 Primary data processing, short read mapping, and single nucleotide polymorphism calling

Raw FASTQ sequences of *P. polytes* mtDNA amplicons (DRA accession numbers: DRA006999 and DRA008115) were subjected to primary data processing. The overall data quality was evaluated by FastQC (Andrews, 2010), and low‐quality 3′‐tails (Phred quality score < 10) were removed automatically using the Perl script DynamicTrim.pl (Cox, Peterson, & Biggs, 2010). The tail‐trimmed sequences were filtered by a custom Perl script to remove reads containing base call failures (N‐bases).

Quality‐filtered sequences were analyzed to estimate the haplotype sequence of the subject mtDNAs using the software described below for short read mapping and variant calling. Sequence reads were aligned to the reference mtDNA sequence of *P. polytes* (15,256 bp; GenBank accession number: NC_024742; Wang, Du, & Li, 2016) using the BWA‐MEM (Burrows–Wheeler alignment with maximal exact matches) algorithm implemented in BWA version 0.7.13 (Li & Durbin, 2010). The sequence alignment/map format files (SAM/BAM) were processed to identify single nucleotide polymorphisms (SNP) using Samtools version 1.3 and Bcftools version 1.3.1 (Li et al., 2009; Narasimhan et al., 2016). Command line options were invoked to skip indel calling and define the sample ploidy as haploid. The mt DNA regions where a SNP quality value ≥30 (error rate < 0.001) was achieved with a sequencing depth of ≥10 of among all samples were used for downstream analyses. The obtained SNP information of mtDNA regions described above was used to replace the corresponding nucleotides of reference mtDNA sequence into those of respective samples to generate a FASTA‐formatted sequence data (DDBJ/EMBL/GenBank accession numbers: LC466203–LC466479) using a custom Perl script.

### 2.5 Molecular phylogenetic and population genetic analyses

To address the demographic history and evolution of Batesian mimicry of *P. polytes* in the Ryukyu Islands, we performed molecular phylogenetic and population genetic analyses. The mtDNA sequences described above were aligned with MAFFT (Katoh & Standley, 2013), and maximum likelihood (ML) and Bayesian phylogenetic analyses were conducted using Treefinder (Jobb, Haeseler, & Strimmer, 2004) and MrBayes 3.2.2. (Ronquist et al., 2012). Appropriate nucleotide substitution models and parameters were estimated by MrModeltest 2.3 (Nylander, 2004) by gene. In the ML analysis, search depth and number of bootstrap replications were set to 2 and 1,000, respectively. In the Bayesian analysis, the Markov chain Monte Carlo process was performed for 1,000,000 generations, and a total of 10,001 trees were sampled. After manual evaluation of the variation and saturation of the likelihood scores, the first 200 trees were discarded, and the final tree and the posterior probabilities of the branches were determined from 9,801 trees. The haplotype network was estimated using the TCS Network method (Templeton, Crandall, & Sing, 1992) by PopART v1.7 (Leigh & Bryant, 2015). Pairwise p‐distances across individuals from multiple islands were calculated using MEGA 7 (Kumar, Stecher, & Tamura, 2016) and visualized using heat maps. A population‐level tree was inferred using the unweighted pair group method with arithmetic mean (UPGMA) method (Sneath & Sokal, 1973) based on genetic distance (Nei's net number of nucleotide differences, *D_A_* [Nei & Li, 1979]). Genetic differentiation (Φ_ST_) among islands was calculated based on *D_A_* divided by the average number of pairwise differences among islands.

Population genetic analyses, including Tajima's *D*‐tests (Tajima, 1989a, 1989b) and mismatch distributions (Rogers & Harpending, 1992), were performed with Arlequin v3.5.2.2 (Excoffier & Lischer, 2010) based on pairwise nucleotide differences as genetic distances with no gamma correction. Tajima's *D* values were assessed by 2,000 permutation tests, and a significant negative value was considered to suggest population expansion in the absence of natural selection (Tajima, 1989a, 1989b). Based on the observed mismatch distributions, the possibility of an instantaneous population expansion was assessed by a generalized nonlinear least square approach, as described in Schneider and Excoffier (1999), and a strong unimodal distribution was considered to suggest a recent expansion (Rogers & Harpending, 1992; Slatkin & Hudson, 1991). The validity of the expansion model was evaluated by the sum of squared deviations between the observed and expected mismatch distributions generated by 5,000 bootstrap resamplings. An expansion time parameter, *t*, was estimated by the equation *t* = *τ*/2*μm*, where *τ* is the mutational timescale estimated from the mismatch distribution, *m* is the DNA sequence length used, and *μ* is the mutation rate. Three known nucleotide substitution rates for the *COI* gene, based on insect mtDNA studies (Vila et al., 2011), were adopted as μ values: 6.5 × 10^−9^ (slow), 7.5 × 10^−9^ (intermediate), and 9.5 × 10^−9^ (fast) substitutions per site and year.

### 2.6 Mimic ratio and advantage index of Batesian mimicry

The MR and the advantage index (AI) of Batesian mimicry of *P. polytes* were defined as follows:

$$\text{MR}=\frac{\#\;\text{mimic}\;P.\;polytes\;\text{females}}{\#\;\text{nonmimic}\;P.\;polytes\;\text{females}+\#\;\text{mimic}\;P.\;polytes\;\text{females}}$$

$$\text{AI}=\frac{\#\;P.\;aristolochiae}{\#\;\text{mimic}\;P.\;polytes\;\text{females}+\#\;P.\;aristolochiae}$$

The definition of MR above is identical to relative abundance (RA) as defined by Sekimura, Fujihashi, and Takechi (2014). The definition of AI was modified from those of Uesugi (2000) and Sekimura et al. (2014) by removing “# nonmimic *P. polytes* females” from the denominator because the nonmimic females (and males) of *P. polytes* do not possess warning signals for avian predators and thus do not affect the mimicry advantage.

### 2.7 Mantel and partial Mantel tests

Possible associations among genetic distance *D_A_*, geographic distance, and difference in MR among islands were assessed by Mantel test (Mantel, 1967) for the five islands with sufficient sample sizes (KIK, OKI, MYK, ISG, and TKT; Figure 1b and Table S1). Associations between differences in MR and differences in environmental factors (described below) among islands were also assessed by Mantel test. For calculation of differences in MR and AI among islands, the numbers of the model species, mimic *P. polytes* females, and nonmimic *P. polytes* females observed in several surveys (Table S2) were totaled for each of the five islands. *D_A_* among islands was calculated by Arlequin v3.5.2.2 (Excoffier & Lischer, 2010) based on the absolute number of substitutions between sequences. Pairwise geographical distances among islands were measured as kilometers between centers of sampling area (i.e., city hall or downtown area) of each island except for OKI where the location of Nakijin village office was used. Climate variables (average temperature, average wind speed, and average rainfall) were transformed into a small number of principal components using R (ver. 3.4.2; the “stats” package), and the absolute values for differences in principal component scores between islands were used as environmental differences (for a summary of the principal components see Table S6). Mantel tests were conducted using Arlequin v3.5.2.2 (Excoffier & Lischer, 2010).

In addition, partial Mantel tests (Smouse, Long, & Sokal, 1986) were conducted to confirm whether AI, rather than other influences, such as historical processes or environmental factors, had shaped the spatial pattern of the MR across the five islands. We estimated the correlation between distance matrices of differences in MR and AI among islands while controlling for the effect of geographic distance, genetic distance *D_A_*, or environmental distances. The partial Mantel test statistic was assessed based on 10,000 permutations of the raw data according to Method 1 in Legendre (2000). These calculations were conducted using a script developed on the R platform (ver. 3.4.2; R Core Team, 2017), which is available from one of the authors (HT).

## 3 RESULTS

### 3.1 General results

We obtained a total of 2,823,806 reads (1,411,903 read pairs), an average of 20,612 ± 35,667 *SD*. reads per individual, ranging from a minimum of 772 to a maximum of 210,558 reads. We used only the regions of mtDNA sequence with high SNP quality (<10^−3^ error rate and ≥10 coverage depth; see Section 2). We obtained mtDNA sequence for the *Cytochrome c oxidase subunit 1* (*COI*) gene and the neighboring region (1,273 bp) for 137 individuals. In addition, for 70 of the 137 individuals, mtDNA sequence was also obtained for the *Cytochrome c oxidase subunit 3* (*COIII*) gene and the neighboring region (442 bp) and the *Cytochrome b* (Cyt *b*) gene and the neighboring regions (568 bp). For a summary of the regions used, see Tables S4 and S5.

### 3.2 Phylogeographic analysis of *P. polytes* in the Ryukyu Islands

**Figure 2:**
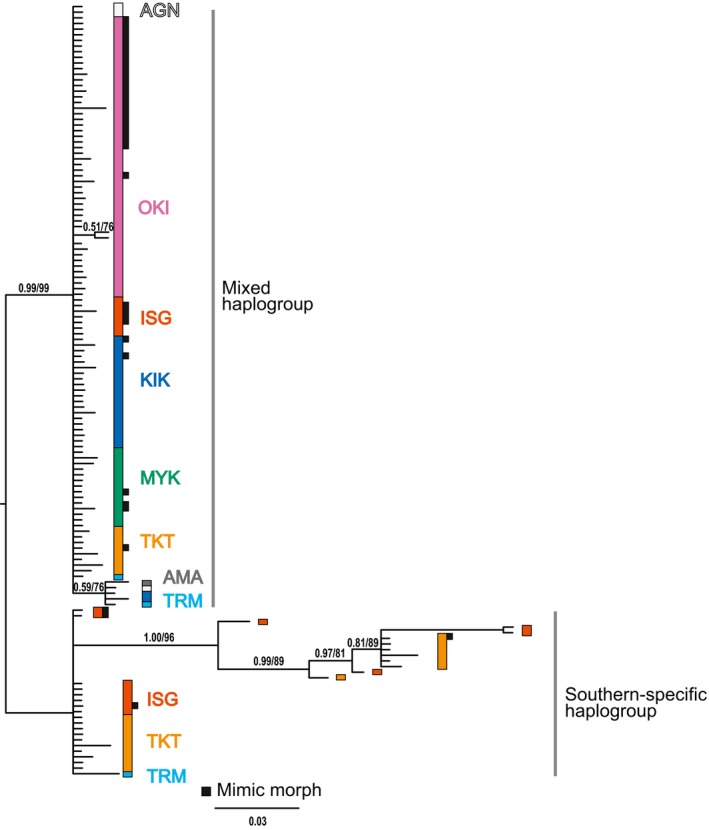
Bayesian/maximum likelihood phylogenic trees and haplotype network of *Papilio polytes* from eight islands of the Ryukyu Islands. Colors indicate the island where the butterfly was collected. Black squares on the right side of branch tips of phylogenetic trees denote mimetic morph samples. Phylogenetic tree of the *COI* gene and neighboring region (1,273 bp) from 137 individuals. The HKY + I nucleotide substitution model was chosen and adapted based on the Bayesian information criterion

Molecular phylogenetic and haplotype network analysis of *P. polytes* consistently indicated two major clades strongly associated with the geographic distribution (Figure 2, Figure S1). One of the major clades contained only individuals collected from southern islands (ISG, TKT, and TRM; the “Southern‐specific haplogroup”) with some deep branching, indicating genetically distant haplotypes. Another clade contained mainly northern individuals (OKI and KIK), along with some southern individuals (the “Mixed haplogroup”), with relatively short branches, indicating closer relationships between the haplotypes within the clade. The mimic‐morph individuals (shown as black squares in Figure 2 and Figure S1a, and with asterisks in Figure S1b) did not form a monophyletic group, but instead were present in both major clades.

**Figure 3:**
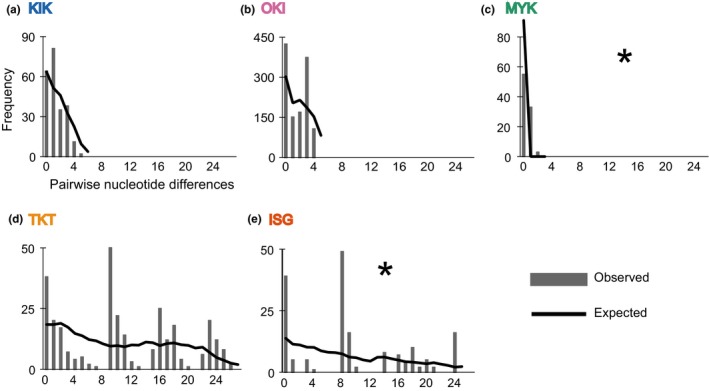
Mismatch distributions in *Papilio polytes* populations of five islands of the Ryukyu Islands. (a) KIK. (b) OKI. (c) MYK. (d) TKT. (e) ISG. Gray bars show observed mismatch distributions based on the absolute number of substitutions in 1,273 bp of the *COI gene* and the neighboring region. Black lines indicate the expected mismatch distribution in the case of demographic expansion based on generalized nonlinear least square fitting (Schneider & Excoffier, 1999). Asterisks denote significant deviation of the expected distribution from the observed distribution, assessed by the sum of squared deviation scores

Heatmap visualization of pairwise genetic distances calculated from 1,273 bp of the *COI* gene and the neighboring region sorted by geographic location (Figure S2a,b) showed essentially the same population genetic structure indicated above. The northern and middle regions of the Ryukyu Islands (KIK, OKI, and MYK) contained closely related individuals. The southern island populations (ISG and TKT) contained several haplotypes that had diverged from each other to some extent. Some of these were more closely related to those from the northern (KIK and OKI) and middle (MYK) islands (light red shading in Figure S2a,b), whereas the remaining haplotypes were genetically distinct, unique haplotypes of the southern islands (ISG and TKT; dark red shading in Figure S2a,b). A UPGMA dendrogram based on 1,273 bp of the *COI* gene and the neighboring region of five populations (KIK, OKI, MYK, ISG, and TKT) implied similar relationships with those depicted by the heatmap. The populations were separated into two distinct clusters concordant with their geographic distribution: southern island populations (ISG and TKT) and northern island populations (KIK, OKI, and MYK) (Figure S2c). Modest but significant genetic differentiation (Φ_ST_) was detected among the populations of these five islands, except for two pairs (ISG–TKT and MYK–KIK) based on 1,273 bp of the *COI* gene and the neighboring region (Table 1). In addition, haplotype frequency‐based genetic differentiation (*F*_ST_) based on 1,273 bp of the *COI* gene and the neighboring region was not detected among these islands (*F*_ST_ ≈ 0.000 in all pairs; data not shown).

**Table 1:** Genetic differentiation (Φ_ST_) among islands

	KIK	OKI	MYK	ISG	TKT
KIK					
OKI	0.418*				
MYK	0.161	0.523*			
ISG	0.412*	0.300*	0.433*		
TKT	0.443*	0.397*	0.457*	0.000	

Note

Genetic differentiation (Φ_ST_) based on Nei's net number of nucleotide differences divided by the average number of pairwise differences among islands.

* Significantly different (*p* < 0.05).

### 3.3 Genetic composition and population expansion of *P. polytes* in the Ryukyu Islands

Our population genetic analyses indicated two types of distinct demographic characteristics of *P. polytes* populations on the five islands of the Ryukyus (KIK, OKI, MYK, TKT, and ISG). The analyses were conducted using the haplotype data for the *COI* gene and the neighboring region (1,273 bp) from 130 individuals from all five islands (min: 14; max: 50; mean: 26 samples per island). Mismatch distribution analysis showed that the northern and middle island populations (KIK, OKI, and MYK) had unimodal distributions, which imply a recent population expansion or rapid turnover (Figure 3a–c), although a significantly negative value for Tajima's *D* was detected only in MYK (Table 2). On the other hand, the southern populations (TKT and ISG) showed multimodal mismatch distributions, which imply a relatively stationary population size with moderate turnover or population equilibrium (Figure 3d,e).

**Table 2:** Neutrality test (Tajima's *D*)

Island	Sample size	No. of haplotypes	S^a^	Pi^b^	Tajima's *D*	Tajima's *D p*‐value
KIK	22	8	7	1.381	−0.899	0.203
OKI	50	8	5	1.664	1.19	0.881
MYK	14	4	3	0.429	−1.671	**0.025**
ISG	19	7	27	9.45	0.88	0.853
TKT	25	12	31	10.947	1.25	0.915

Note

Bold type *p*‐values indicate statistical significance (*p* < 0.05).

a Number of segregating sites within the sample.

b Mean number of differences between all pairs of haplotypes within the sample.

Population expansion times were estimated for the KIK, OKI, and TKT samples, where the expected mismatch distributions from a sudden expansion model did not significantly deviate from the observed distributions (Table 3). The inferred timing of population expansion was relatively recent in the northern islands (31,000 to 45,000 years ago for KIK and 151,000 to 221,000 years ago for OKI), whereas on the southern island (TKT) the expansion was estimated to have occurred before that in the northern islands (0.878 to 1.283 million years ago).

**Table 3:** Mismatch distribution analyses

Island	Tau	SSD	*p*	Expansion time (*L*) (MYA)	Expansion time (*M*) (MYA)	Expansion time (*H*) (MYA)	Elevation (m)^a^	Age of island^b^
KIK	0.742	0.008	0.288	0.045	0.039	0.031	214	~0.85 MYA
OKI	3.656	0.044	0.211	0.221	0.191	0.151	503	>2.0 MYA (originally a continental margin arc)
MYK	0.500	0.005	0.000	0.030	0.026	0.021	115	
ISG	23.693	0.115	0.013	1.432	1.241	0.980	526	
TKT	21.234	0.040	0.198	1.283	1.112	0.878	33	

Note

Tau is the moment estimator of the time to expansion (age of expansion). SSD indicates the sum of squared deviations between the observed and the expected mismatch distribution, assuming a sudden population expansion as a test statistic. *p* (Sim. SSD ≥ Obs. SSD)‐values < 0.05 indicate significant deviation from the expected distribution, assuming a sudden population expansion model. Expansion times (*L*), (*M*), and (*H*) were calculated based on approximate mutation rate (= substitution rate) *μ*, with slow, intermediate, and fast rates of 6.5 × 10^−9^, 7.5 × 10^−9^, and 9.5 × 10^−9^ substitutions per site and year, respectively.

Abbreviation: MYA, million years ago.

a Data from the Geospatial Information Authority of Japan (2018).

b Data from Osozawa et al. (2012).

### 3.4 Mantel and partial Mantel tests

**Figure 4:**
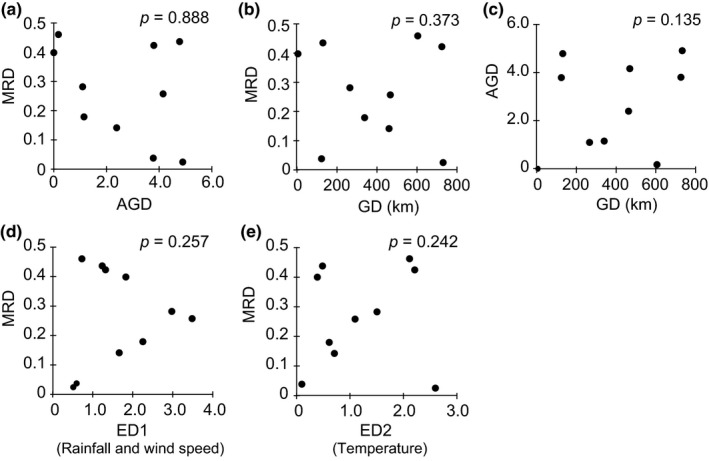
Mantel test for (a) mimic ratio difference (MRD) and average genetic distance (AGD), (b) MRD and geographic distance (GD), (c) AGD and GD, (d) and (e) MRD and environmental distances (ED1 and ED2) among five Ryukyu Islands. AGD was calculated using Nei's net number of nucleotide differences (*D_A_*). ED1 and ED2 are the difference between PC1 and PC2, generated using various climate variables. ED1 mainly reflects differences in average rainfall and average wind speed among islands. ED2 mainly reflects differences in average temperature among islands (see Table S6). Datapoints represent pairwise combinations of the five islands (KIK, Kikai; OKI, Okinawa; MYK, Miyako; ISG, Ishigaki; TKT, Taketomi). The *p*‐value shown in each panel is for the Mantel test

**Figure 5:**
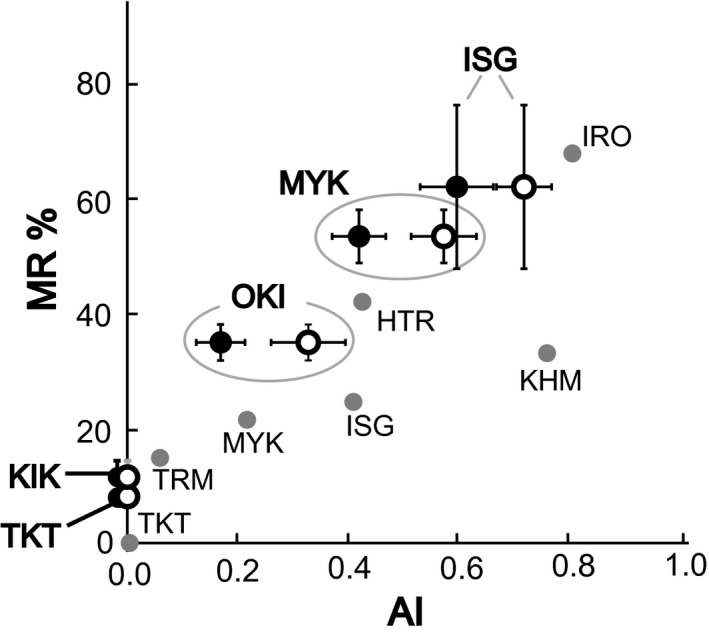
Relationship between mimic ratio (MR) and advantage index (AI) of Batesian mimicry in *Papilio polytes*. Datapoints derived from our survey conducted 2014–2017 are indicated by white and black dots. The white dots correspond to AI values calculated using the method of the present study, whereas the black dots correspond to AI values calculated using the method of Uesugi (2000) and Sekimura et al. (2014). White and black dots represent the mean values for each island. Error bars show standard error calculated using data collected during several surveys conducted on different days (for raw data, see Table S2). Gray dots indicate datapoints based on the 1982 survey (Uesugi, 2000) and the AI calculation method of Uesugi (2000) and Sekimura et al. (2014)

Regarding genetic distance (*D_A_*), geographic distance, and MR differences among *P. polytes* populations, no significant association was detected among the five islands of the Ryukyu Islands (KIK, OKI, MYK, TKT, and ISG; Figure 1b), based on the Mantel test (Figure 4a–c). Furthermore, the Mantel test confirmed that neither of the environmental distances was associated with MR differences among the five islands (Figure 4d,e). On the other hand, the partial Mantel tests confirmed a significant association between MR differences and AI differences among the five islands while controlling for the effect of either geographic distance (GD), genetic distance (AGD), or environmental distances (*p* < 0.01 in all cases; for a summary of the results, see Table 4; Figure 5, white dots). The results were substantially the same when a previous method for calculation of AI (Sekimura et al., 2014; Uesugi, 2000) was applied (*p* < 0.05 in all cases, Table 4; Figure 5, black dots). MR and AI were also found to be correlated in a previous survey of seven islands conducted in 1982 (Uesugi, 2000; Figure 5, gray dots), indicating a striking correspondence between *P. polytes* mimicry and the density of the model species (*P. aristolochiae*) in the Ryukyu Islands for several decades.

**Table 4:** Partial Mantel tests

Association	Controlled factor	*r* a	*p*‐Value
MRD–AID^b^	GD	0.913	<0.001
	AGD	0.898	<0.001
	ED1	0.912	0.001
	ED2	0.923	<0.001
MRD–AID_Uesugi_ c	GD	0.742	0.011
	AGD	0.701	0.019
	ED1	0.762	0.010
	ED2	0.771	0.008

Note

Estimation of the correlation between distance matrices of differences in mimic ratio (MR) and advantage index (AI) (MRD and AID) among islands, controlling for the effects of geographic distance (GD), genetic distance (AGD; *D_A_* [Nei & Li, 1979]), environmental distance 1 (ED1), or environmental distance 2 (ED2). ED1 and 2 are the difference between PC1 and PC2, generated using various climate variables. ED1 mainly reflects differences in average rainfall and average wind speed among islands. ED2 mainly reflects differences in average temperature among islands.

a Partial correlation coefficient.

b AI and AID were calculated based on the definition described in the Section 2.

c AI and AID were calculated based on the definition described in previous studies (Sekimura et al., 2014; Uesugi, 2000).

## 4 DISCUSSION

We hypothesized that in *P. polytes* populations the frequency of the Batesian mimicry morph is limited by the abundance of the model species (*P. aristolochiae*) and investigated populations of these two butterflies on eight of the Ryukyu Islands, Japan (Figure 1). We found that the mimic ratio and the model abundance (MR and AI, respectively) are positively associated (Figure 5), although each island exhibited unique MR and AI values, independent of geographical position (Figure 1b). We confirmed the significant association between MR differences and AI differences among the five islands, while controlling for the effect of either GD, AGD, or environmental distances (for a summary of environmental factors see Section 2, Table S6, and Table 4; Figure 5). Accordingly, we propose that Batesian mimicry in *P. polytes* is maintained by negative frequency‐dependent selection shaped by model species abundance and that the MR on each island has adjusted to the local abundance of the model species through NFDS. These views have been proposed previously (Barrett, 1976; Kunte, 2009; Turner, 1978); however, the present study provides the first systematic evidence from field surveys.

We also investigated the phylogeographic history of *P. polytes* in the Ryukyu Islands to examine whether neutral forces such as phylogenetic constraint or isolation by distance affected the distribution of mimicry patterns (Figures 2, 3, 4, 5, Figures S1 and S2). Molecular phylogenetic and haplotype network analysis of mtDNA sequences indicates that the populations fall into two major clades corresponding to geographic distribution, not mimetic patterns (Figures 2 and S1). One of the major clades is composed only of individuals from the southern islands (ISG, TKT, and TRM; see Figure 2, “Southern‐specific haplogroup”) and includes genetically diverse, long‐branched haplotypes (Figures 2 and S1). On the other hand, the remaining clade is comprised of individuals from all islands investigated and contains more closely related, short‐branched haplotypes (Figures 2 and S1, “Mixed haplogroup”). This phylogenetic pattern implies that the southern islands are a major source of genetic diversity (e.g., ISG, TKT, TRM; see Figures 1b and 2, Figure S1), and the middle and northern island populations (MYK, KIK, OKI, and AGN) likely arose by relatively recent migration and colonization. This view is supported by several population genetic characteristics (Figure 3, Tables 2 and 3), which suggest a relatively recent population expansion in the middle and northern islands (KIK, OKI, and MYK), and stable population persistence in the southern islands (TKT and ISG), based on mismatch distribution and Tajima's *D* analyses.

Taken together, the above findings suggest that the MR of *P. polytes* populations has adjusted to the conditions on each island, whereas the migration and gene flow have occurred to some extent among the Ryukyu Islands. The supposed migration from south to north (see Figures 1b and 2, Figure S1) seems probable, given the low‐level jet stream that is present in this area in spring and summer (Japan Meteorological Agency, 2016). These south–north wind patterns are reported to promote passive migration of other flying insects, such as rice planthoppers *Sogatella furcifera* Horváth and *Nilaparvata lugens* Stål (Kishimoto, 1976; Seino, Shiotsuki, Oya, & Hirai, 1987); they likely affect the *P. polytes* habitats also. This view is supported by our results implying recent population turnover and/or expansion in the middle and northern islands (Figure 3, Tables 2 and 3) and the slight to modest genetic differentiation observed among the island populations investigated (*F*_ST_  ≈ 0.000 in all pairs and Table 1). In spite of the migration and gene flow, maintenance of a larger proportion of mimetic females is allowed by the greater local abundance of the model species (see Figures 1b and 5, Table 4), and the observed MR differentiation cannot be explained by genetic, geographic, or environmental distance (see Figure 4, Table S6). Based on these findings, we propose that the strong predation pressure on this butterfly (Katoh et al., 2017), rather than isolation by distance or other neutral processes, has shaped and maintained the Batesian mimicry patterns of *P. polytes* observed in the Ryukyu Islands.

It is generally difficult to demonstrate predation pressure on prey species (*P. polytes* in this case) in the wild or in field surveys; however, our preliminary data for beak marks on *P. polytes* indicate predation by birds. The strength of the predation pressure can be estimated from beak marks (e.g., Ohsaki, 1995), and that on mimetic females of *P. polytes* seems to depend upon the abundance of the model species in the Ryukyu Islands (data not shown). Furthermore, Katoh et al. (2017) reported recent changes in wing coloration pattern in mimetic females of *P. polytes* with emergence of new variations of mimetic patterns of the same form in the Ryukyu Islands, suggesting that *P. polytes* appears to be under constant predation pressure. These data support our view that predation pressure combined with model species abundance determines the MR of *P. polytes* females.

According to our population genetic analysis, the KIK (Kikai) population of *P. polytes* was estimated to have been established in the relatively recent past (31,000 to 45,000 years ago; Table 3), after which it underwent population expansion (Figure 3). This implication does not conflict with the relatively young age of KIK Island, estimated based on geological data to have arisen about 0.85 million years ago, compared with the other islands investigated (Table 3; Osozawa et al., 2012). In addition, the significantly negative value of Tajima's *D* (Table 2) estimated for the MYK (Miyako) population suggests rapid turnover and/or recent colonization. This view is compatible with the known distribution patterns of other butterflies; several species are distributed across all of the Ryukyu Islands except MYK (Satonaka, 2014). On MYK, which is characteristically flat with a maximum altitude of 115 m, butterflies, including *P. polytes,* might have become locally extinct following island subsidence or other catastrophic events (Table 3). Although TKT (Taketomi) Island also has a lower elevation (33 m; Table 3), the TKT population was estimated to have persisted at a stable size (Figure 3 and Table 3). TKT is located close to ISG (Ishigaki) (Figure 1b), and TKT could easily be recolonized by the ISG population (Figure 2). This view is supported by the absence of significant genetic differentiation between ISG and TKT (Table 1). Altogether, the consistent patterns observed across several types of analysis support these conclusions.

In future works, massive and comprehensive SNP analysis of the nuclear genome could be used to confirm the evolutionary history of the *P. polytes* populations examined in this study. Restriction site‐associated DNA sequencing (RAD‐seq), multiplexed intersimple sequence repeat genotyping by sequencing (MIG‐seq) (Suyama & Matsuki, 2015), or other methods could be used. In addition, an ecogenomic analysis of the *doublesex* (*dsx*) gene could provide a useful perspective, because the *dsx* gene is thought to be responsible for mimicry polymorphism in *P. polytes* (Kunte et al., 2014; Nishikawa et al., 2015; Zhang, Westerman, Nitzany, Palmer, & Kronforst, 2017) and may play a key role in the rapid evolution of *P. polytes* mimicry in the Ryukyu Islands. Molecular analysis to detect the signatures of selection in the *dsx* gene would also be worthy of future study.

## CONFLICT OF INTEREST

None declared.

## AUTHOR CONTRIBUTIONS

KT and KT‐S designed the study. KT‐S, YS, MK, and EK performed the research. YS, KT‐S, EK, and HT analyzed data. All authors contributed to writing the manuscript.

## Data Availability

Sequencing reads are available at the DDBJ Sequence Read Archive (DRA) under the accession numbers DRA006999 and DRA008115. FASTA‐formatted sequence data reported are available in the DDBJ/EMBL/GenBank databases under the accession numbers LC466203–LC466479.

## References

[ece35182-bib-0001] Ackermann, R. R. , & Cheverud, J. M. (2004). Detecting genetic drift versus selection in human evolution. Proceedings of the National Academy of Sciences of the United States of America, 101(52), 17946–17951. 10.1073/pnas.0405919102 15604148 PMC539739

[ece35182-bib-0002] Andrews, S. (2010). FastQC: A quality control tool for high throughput sequence data. Retrieved from http://www.bioinformatics.babraham.ac.uk/projects/fastqc

[ece35182-bib-0003] Barrett, J. A. (1976). The maintenance of non‐mimetic forms in a dimorphic Batesian mimic species. Evolution, 30(1), 82–85. 10.1111/j.1558-5646.1976.tb00883.x 28565053

[ece35182-bib-0004] Bates, H. W. (1862). Contributions to an insect fauna of the Amazon Valley (Lepidoptera: Heliconidae). Transactions of the Linnean Society of London, 23(3), 495–556.

[ece35182-bib-0005] Burns, J. M. (1966). Preferential mating versus mimicry: Disruptive selection and sex‐limited dimorphism in *Papilio glaucus* . Science, 153(3735), 551–553. 17830374 10.1126/science.153.3735.551

[ece35182-bib-0006] Clarke, C. A. , & Sheppard, P. M. (1972). The genetics of the mimetic butterfly* Papilio polytes* L. Philosophical Transactions of the Royal Society B: Biological Sciences, 263(855), 431–458. 10.1098/rstb.1972.0006 4402450

[ece35182-bib-0007] Cook, S. E. , Vernon, J. G. , Bateson, M. , & Guilford, T. (1994). Mate choice in the polymorphic African swallowtail butterfly, *Papilio dardanus*: Male‐like females may avoid sexual harassment. Animal Behaviour, 47(2), 389–397. 10.1006/anbe.1994.1053

[ece35182-bib-0008] Cott, H. B. (1940). Adaptive Coloration in Animals. Methuen.

[ece35182-bib-0009] Cox, M. P. , Peterson, D. A. , & Biggs, P. J. (2010). SolexaQA: At‐a‐glance quality assessment of Illumina second‐generation sequencing data. BMC Bioinformatics, 11, 485 10.1186/1471-2105-11-485 20875133 PMC2956736

[ece35182-bib-0010] Edmunds, M. (1974). Defence in animals. London, UK: Longman.

[ece35182-bib-0011] Excoffier, L. , & Lischer, H. E. (2010). Arlequin suite ver 3.5: A new series of programs to perform population genetics analyses under Linux and Windows. Molecular Ecology Resources, 10(3), 564–567. 21565059 10.1111/j.1755-0998.2010.02847.x

[ece35182-bib-0012] Ford, E. B. (1975). Ecological genetics, 4th ed. London, UK: Methuen.

[ece35182-bib-0013] Geospatial Information Authority of Japan (2018). GSI Maps. Retrieved from http://www.gsi.go.jp/ENGLISH/page_e30233.html

[ece35182-bib-0014] Japan Meteorological Agency (2016). Weather observation data search (in Japanese). Retrieved from http://www.data.jma.go.jp/obd/stats/etrn/index.php

[ece35182-bib-0015] Jobb, G. , Haeseler, A. , & Strimmer, K. (2004). TREEFINDER. Retrieved from http://www.treefinder.de/

[ece35182-bib-0016] Katoh, K. , & Standley, D. M. (2013). MAFFT multiple sequence alignment software version 7: Improvements in performance and usability. Molecular Biology and Evolution, 30(4), 772–780. 10.1093/molbev/mst010 23329690 PMC3603318

[ece35182-bib-0017] Katoh, M. , Tatsuta, H. , & Tsuji, K. (2017). Rapid evolution of a Batesian mimicry trait in a butterfly responding to arrival of a new model. Scientific Reports, 7, 6369 10.1038/s41598-017-06376-9 28743998 PMC5527021

[ece35182-bib-0018] Kishimoto, R. (1976). Synoptic weather conditions inducing long‐distance immigration of planthoppers, *Sogatella furcifera* Horvath and *Nilaparvata lugens* Stål. Ecological Entomology, 1(2), 95–109.

[ece35182-bib-0019] Kumar, S. , Stecher, G. , & Tamura, K. (2016). MEGA7: Molecular Evolutionary Genetics Analysis Version 7.0 for bigger datasets. Molecular Biology and Evolution, 33(7), 1870–1874. 10.1093/molbev/msw054 27004904 PMC8210823

[ece35182-bib-0020] Kunte, K. (2009). Female‐limited mimetic polymorphism: A review of theories and a critique of sexual selection as balancing selection. Animal Behaviour, 78(5), 1029–1036. 10.1016/j.anbehav.2009.08.013

[ece35182-bib-0021] Kunte, K. , Zhang, W. , Tenger‐Trolander, A. , Palmer, D. H. , Martin, A. , Reed, R. D. , … Kronforst, M. R. (2014). Doublesex is a mimicry supergene. Nature, 507(7491), 229–232. 24598547 10.1038/nature13112

[ece35182-bib-0023] Legendre, P. (2000). Comparison of permutation methods for the partial correlation and partial Mantel tests. Journal of Statistical Computation and Simulation, 67(1), 37–73. 10.1080/00949650008812035

[ece35182-bib-0024] Leigh, J. W. , & Bryant, D. (2015). PopART: Full‐feature software for haplotype network construction. Methods in Ecology and Evolution, 6(9), 1110–1116.

[ece35182-bib-0025] Li, H. , & Durbin, R. (2010). Fast and accurate long‐read alignment with Burrows‐Wheeler transform. Bioinformatics, 26(5), 589–595. 10.1093/bioinformatics/btp698 20080505 PMC2828108

[ece35182-bib-0026] Li, H. , Handsaker, B. , Wysoker, A. , Fennell, T. , Ruan, J. , & Homer, N. , … 1000 Genome Project Data Processing Subgroup (2009). The sequence alignment/map format and SAMtools. Bioinformatics, 25(16), 2078–2079. 10.1093/bioinformatics/btp352 19505943 PMC2723002

[ece35182-bib-0027] Mallet, J. , & Joron, M. (1999). Evolution of diversity in warning color and mimicry: Polymorphisms, shifting balance, and speciation. Annual Review of Ecology and Systematics, 30, 201–233. 10.1146/annurev.ecolsys.30.1.201

[ece35182-bib-0028] Mantel, N. (1967). The detection of disease clustering and a generalized regression approach. Cancer Research, 27(2 Part 1), 209–220. 6018555

[ece35182-bib-0029] Narasimhan, V. , Danecek, P. , Scally, A. , Xue, Y. , Tyler‐Smith, C. , & Durbin, R. (2016). BCFtools/RoH: A hidden Markov model approach for detecting autozygosity from next‐generation sequencing data. Bioinformatics, 32(11), 1749–1751. 10.1093/bioinformatics/btw044 26826718 PMC4892413

[ece35182-bib-0030] Nei, M. , & Li, W. H. (1979). Mathematical model for studying genetic variation in terms of restriction endonucleases. Proceedings of the National Academy of Sciences of the United States of America, 76, 5269–5273. 291943 10.1073/pnas.76.10.5269 PMC413122

[ece35182-bib-0031] Nishikawa, H. , Iijima, T. , Kajitani, R. , Yamaguchi, J. , Ando, T. , Suzuki, Y. , … Fujiwara, H. (2015). A genetic mechanism for female‐limited Batesian mimicry in *Papilio* butterfly. Nature Genetics, 47(4), 405–409. 10.1038/ng.3241 25751626

[ece35182-bib-0032] Nylander, J. A. A. (2004). MrModeltest v2. Program distributed by the author. Evolutionary Biology Centre, Uppsala University.

[ece35182-bib-0033] Ohsaki, N. (1995). Preferential predation of female butterflies and the evolution of batesian mimicry. Nature, 378, 173–175. 10.1038/378173a0

[ece35182-bib-0034] Ohsaki, N. (2005). A common mechanism explaining the evolution of female‐limited and both‐sex Batesian mimicry in butterflies. Journal of Animal Ecology, 74(4), 728–734. 10.1111/j.1365-2656.2005.00972.x

[ece35182-bib-0035] Osozawa, S. , Shinjo, R. , Armid, A. , Watanabe, Y. , Horiguchi, T. , & Wakabayashi, J. (2012). Palaeogeographic reconstruction of the 1.55 Ma synchronous isolation of the Ryukyu Islands, Japan, and Taiwan and inflow of the Kuroshio warm current. International Geology Review, 54(12), 1369–1388.

[ece35182-bib-0036] R Core Team (2017). R: A language and environment for statistical computing. Vienna, Austria: R Foundation for Statistical Computing Retrieved from https://www.R-project.org/

[ece35182-bib-0037] Rettenmeyer, C. W. (1970). Insect mimicry. Annual Review of Entomology, 15(1), 43–74. 10.1146/annurev.en.15.010170.000355

[ece35182-bib-0038] Rogers, A. R. , & Harpending, H. (1992). Population growth makes waves in the distribution of pairwise genetic divergences. Molecular Biology and Evolution, 9(3), 552–569. 1316531 10.1093/oxfordjournals.molbev.a040727

[ece35182-bib-0039] Ronquist, F. , Teslenko, M. , Van Der Mark, P. , Ayres, D. L. , Darling, A. , Höhna, S. , … Huelsenbeck, J. P. (2012). MrBayes 3.2: Efficient Bayesian phylogenetic inference and model choice across a large model space. Systematic Biology, 61(3), 539–542. 22357727 10.1093/sysbio/sys029 PMC3329765

[ece35182-bib-0040] Ruxton, G. D. , Sherratt, T. N. , & Speed, M. P. (2004). Avoiding attack. Oxford, UK: Oxford University Press.

[ece35182-bib-0041] Satonaka, M. (2014). Distribution, geographic variation of butterflies in Kyushu, Ryukyu and Taiwan, and habitat of those butterflies. Yadoriga, 242, 2–11. (In Japanese.)

[ece35182-bib-0042] Schneider, S. , & Excoffier, L. (1999). Estimation of past demographic parameters from the distribution of pairwise differences when the mutation rates vary among sites: Application to human mitochondrial DNA. Genetics, 152(3), 1079–1089. 10388826 10.1093/genetics/152.3.1079 PMC1460660

[ece35182-bib-0043] Seino, H. , Shiotsuki, Y. , Oya, S. , & Hirai, Y. (1987). Prediction of long distance migration of rice planthoppers to Northern Kyushu considering low‐level jet stream. Journal of Agricultural Meteorology, 43(3), 203–208. 10.2480/agrmet.43.203

[ece35182-bib-0044] Sekimura, T. , Fujihashi, Y. , & Takechi, Y. (2014). A model for population dynamics of the mimetic butterfly* Papilio polytes* in the Sakishima Islands, Japan. Journal of Theoretical Biology, 361, 133–140. 10.1016/j.jtbi.2014.06.029 25036440

[ece35182-bib-0045] Simon, C. , Frati, F. , Beckenbach, A. , Crespi, B. , Liu, H. , & Flook, P. (1994). Evolution, weighting, and phylogenetic utility of mitochondrial gene sequences and compilation of conserved polymerase chain reaction primers. Annals of the Entomological Society of America, 87(6), 651–701.

[ece35182-bib-0046] Slatkin, M. , & Hudson, R. R. (1991). Pairwise comparisons of mitochondrial DNA sequences in stable and exponentially growing populations. Genetics, 129(2), 555–562. 1743491 10.1093/genetics/129.2.555 PMC1204643

[ece35182-bib-0047] Smouse, P. E. , Long, J. C. , & Sokal, R. R. (1986). Multiple regression and correlation extensions of the Mantel test of matrix correspondence. Systematic Zoology, 35(4), 627–632. 10.2307/2413122

[ece35182-bib-0048] Sneath, P. H. A. , & Sokal, R. R. (1973). Numerical taxonomy. San Francisco, CA: Freeman.

[ece35182-bib-0049] Song, H. , Buhay, J. E. , Whiting, M. F. , & Crandall, K. A. (2008). Many species in one: DNA barcoding overestimates the number of species when nuclear mitochondrial pseudogenes are coamplified. Proceedings of the National Academy of Sciences of the United States of America, 105(36), 13486–13491. 10.1073/pnas.0803076105 18757756 PMC2527351

[ece35182-bib-0050] Suyama, Y. , & Matsuki, Y. (2015). MIG‐seq: An effective PCR‐based method for genome‐wide single‐nucleotide polymorphism genotyping using the next‐generation sequencing platform. Scientific Reports, 5, 16963 10.1038/srep16963 26593239 PMC4655332

[ece35182-bib-0051] Tajima, F. (1989a). Statistical method for testing the neutral mutation hypothesis by DNA polymorphism. Genetics, 123(3), 585–595. 2513255 10.1093/genetics/123.3.585 PMC1203831

[ece35182-bib-0052] Tajima, F. (1989b). The effect of change in population size on population DNA polymorphism. Genetics, 123(3), 597–601. 2599369 10.1093/genetics/123.3.597 PMC1203832

[ece35182-bib-0053] Templeton, A. R. , Crandall, K. A. , & Sing, C. F. (1992). A cladistic analysis of phenotypic associations with haplotypes inferred from restriction endonuclease mapping and DNA sequence data. III. Cladogram estimation. Genetics, 132(2), 619–633. 1385266 10.1093/genetics/132.2.619 PMC1205162

[ece35182-bib-0054] Turner, J. R. G. (1978). Why male butterflies are non‐mimetic: Natural selection, sexual selection, group selection, modification and sieving. Biological Journal of the Linnean Society, 10(4), 385–432. 10.1111/j.1095-8312.1978.tb00023.x

[ece35182-bib-0055] Uesugi, K. (2000). Avoiding attack in adult butterflies In Ohsaki N. (Ed.), The natural history of butterflies (pp. 106–123). Sapporo, Japan: Hokkaido University Press. (In Japanese.)

[ece35182-bib-0056] Vane‐Wright, R. I. (1984). The role of pseudosexual selection in the evolution of butterfly colour patterns. In Vane‐Wright R. I., & Ackery P. R. (Eds.), The biology of butterflies (pp. 251–253). Cambridge, MA:Academic Press.

[ece35182-bib-0057] Vila, R. , Bell, C. D. , Macniven, R. , Goldman‐Huertas, B. , Ree, R. H. , Marshall, C. R. , … Pierce, N. E. (2011). Phylogeny and palaeoecology of *Polyommatus* blue butterflies show Beringia was a climate‐regulated gateway to the New World. Proceedings of the Royal Society of London B: Biological Sciences, 278(1719), 2737–2744. 10.1098/rspb.2010.2213 PMC3145179 21270033

[ece35182-bib-0058] Wallace, A. R. (1865). On the phenomena of variation and geographical distribution as illustrated by the Papilionidae of the Malayan region. Transactions of the Linnean Society of London, 25, 1–71.

[ece35182-bib-0059] Wang, L. , Du, X. J. , & Li, X. F. (2016). The complete mitogenome of the Common Mormon *Papilio polytes* (Insecta: Lepidoptera: Papilionoidea). Mitochondrial DNA Part A, 27(2), 1269–1270. 10.3109/19401736.2014.945550 25090394

[ece35182-bib-0060] Wickler, W. (1968). Mimicry in plants and animals. New York: McGraw‐Hill.

[ece35182-bib-0061] Wright, S. (1943). Isolation by distance. Genetics, 28(2), 114–138. 17247074 10.1093/genetics/28.2.114 PMC1209196

[ece35182-bib-0062] Zhang, W. , Westerman, E. , Nitzany, E. , Palmer, S. , & Kronforst, M. R. (2017). Tracing the origin and evolution of supergene mimicry in butterflies. Nature Communications, 8(1), 1269 10.1038/s41467-017-01370-1 PMC5677128 29116078

