# Supplementary material for: Evidence for frequency‐dependent selection maintaining polymorphism in the Batesian mimic Papilio polytes in multiple islands in the Ryukyus, Japan
Source: Ecol Evol. 2019 Apr 24;9(10):5991–6002. doi: 10.1002/ece3.5182 (PMC6540699; doi:10.1002/ece3.5182)
Supplement: Supplementary file 1 [file ECE3-9-5991-s001.docx]

**Supporting information Figures**

**Evidence for frequency-dependent selection maintaining polymorphism in the Batesian mimic *Papilio polytes* in multiple islands in the Ryukyus, Japan**

Kaori Tsurui-Sato^1,*,†^, Yukuto Sato^1,†^, Emi Kato^2,†^, Mitsuho Katoh^2, 3,†^, Ryosuke Kimura^4^, Haruki Tatsuta^2, 3^, Kazuki Tsuji^2, 3, *^

1. Center for Strategic Research Project, University of the Ryukyus, Okinawa, 903-0213, Japan

2. Department of Agro-Environmental Sciences, Faculty of Agriculture, University of the Ryukyus, Okinawa, 903-0213, Japan

3. The United Graduate School of Agricultural Sciences, Kagoshima University, Korimoto 1-21-24, Kagoshima, 890-8580, Japan

4. Department of Human Biology and Anatomy, Graduate School of Medicine, University of the Ryukyus, Okinawa, 903-0215, Japan

**^†^These authors contributed equally to this work.**

***To whom correspondence should be addressed:**

Kaori Tsurui-Sato

Tel: +81-98-886-4191; Fax: +81-098-887-7188; Email: tsuruikaori@gmail.com

Kazuki Tsuji

Tel: +81-98-895-8797; Fax: +81-98-895-8797; Email: tsujik@agr.u-ryukyu.ac.jp


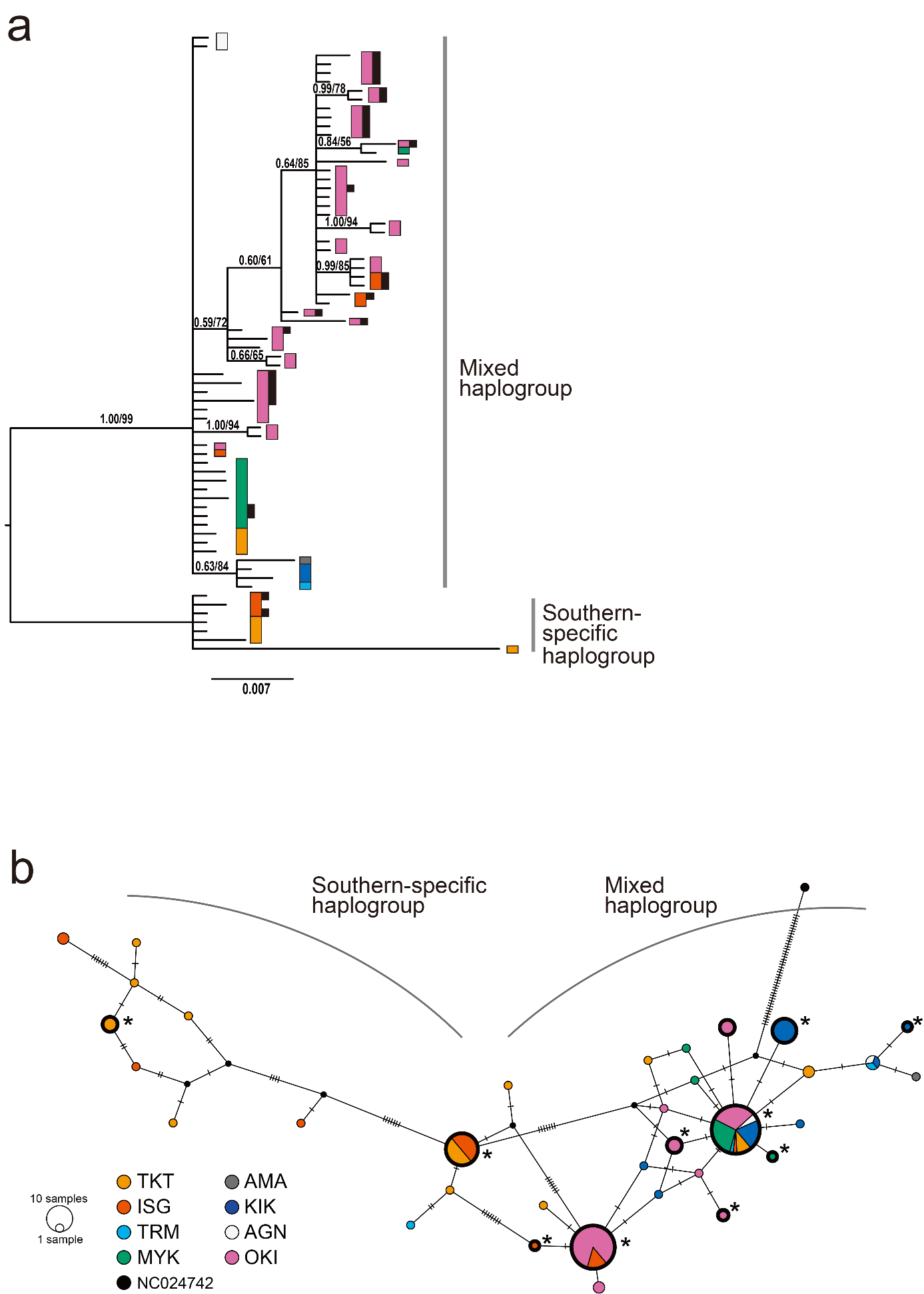


**Supporting information Figure S1. Bayesian/maximum-likelihood phylogenic trees and haplotype network of *Papilio polytes* from eight islands of the Ryukyu Islands*.***

***.*** Colors indicate the island where the butterfly was collected. Black squares on the right side of branch tips of phylogenetic trees denote mimetic morph samples. (a) Phylogenetic tree of concatenated data of COI, COIII, and Cyt *b* genes and their neighboring regions (1,273, 442, and 568 bp, respectively) from 70 individuals. The HKY substitution model was chosen for the latter two regions. Values assigned to each node of the tree represent posterior probability and bootstrap support (values < 0.50 and 50 were omitted, respectively). (b) Haplotype network of the COI region (1,273 bp). Circle size indicates the number of individuals. Sample geographic origin is indicated by color. Asterisks indicate network nodes that include more than one mimetic female.

**
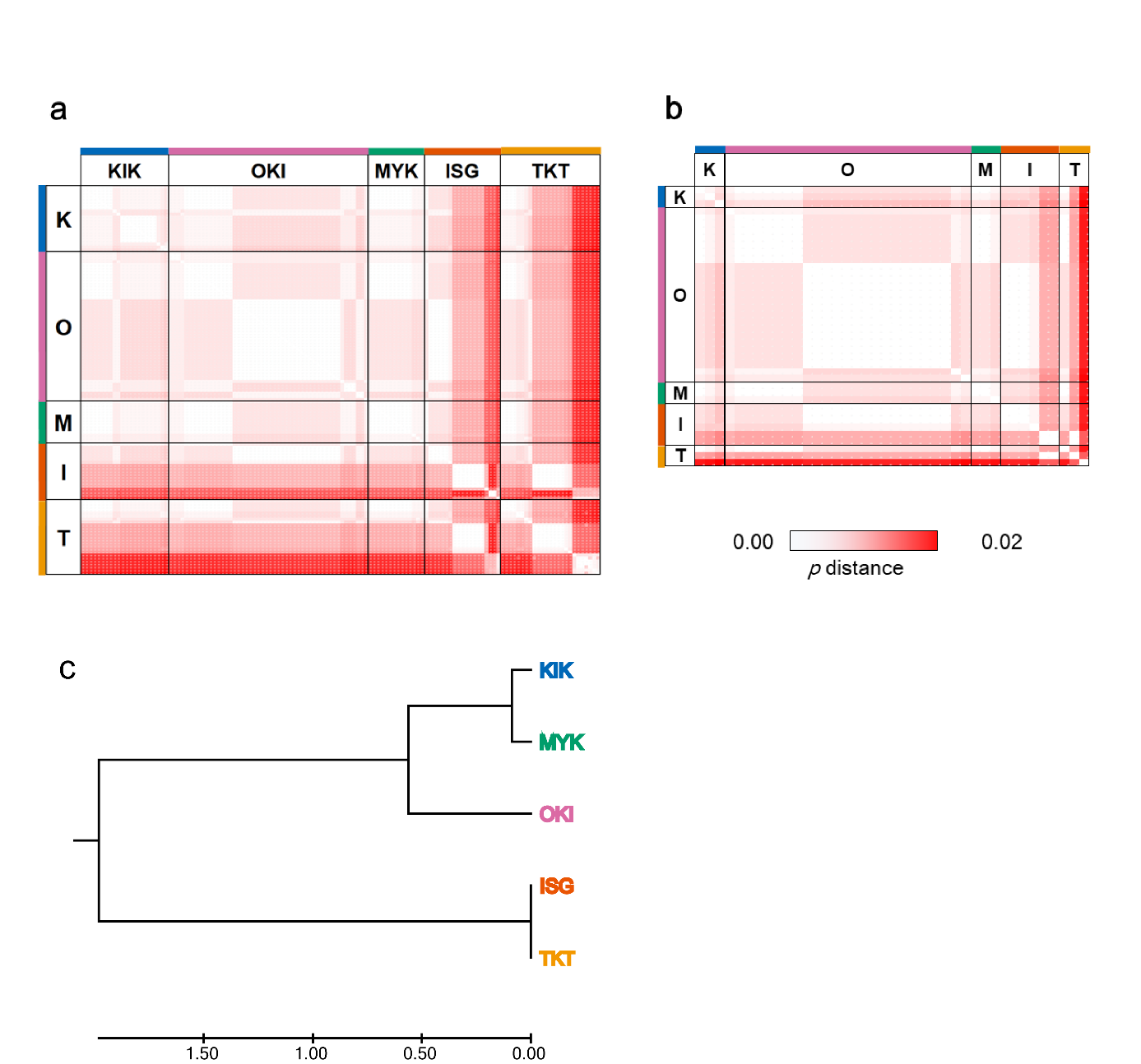
**

**Supporting information Figure S2. Heatmap of pairwise genetic distance and phylogenetic tree of populations across individuals from five islands of the Ryukyu Islands.** (a) All individuals (n = 130). (b) Mimetic females (n = 40). Color bars indicate the island on which the butterfly was collected, and columns and rows denote each individual. Matrix shading shows pairwise *p* distance across 130 and 40 individuals for (a) and (b), respectively, calculated using the absolute number of substitutions in 1,273 bp of the *COI gene* and the neighboring region. (c) The population-level tree among five islands inferred using the UPGMA method (Sneath & Sokal 1973) based on ***D_A_*** calculated from 1,273 bp of the *COI* gene and the neighboring region (n = 130).
